# Operative Management of Spinal Deformity Secondary to Hajdu-Cheney Syndrome

**DOI:** 10.7759/cureus.17334

**Published:** 2021-08-20

**Authors:** Cody J Falls, Paul S Page, James A Stadler

**Affiliations:** 1 Orthopedic Surgery, University of Wisconsin School of Medicine and Public Health, Madison, USA; 2 Neurological Surgery, University of Wisconsin School of Medicine and Public Health, Madison, USA

**Keywords:** hajdu-cheney syndrome, spine, deformity, craniocervical, scoliosis, pediatric, metabolic bone disease, hcs, skeletal, suture diastasis

## Abstract

Hajdu-Cheney syndrome (HCS) is an exceedingly rare disease with fewer than 100 cases described in the medical literature. It is most strongly associated with a defect in the transmembrane protein NOTCH2. Though the exact mechanism in humans is not yet known, the defect results in various skeletal abnormalities including severe osteoporosis placing these patients at high risk for progressive spinal deformity. Due to various common syndromic features including ligamentous laxity, increased osteoclast activity, skeletal malformations, patency of cranial sutures, and the aforementioned severe osteoporosis, these patients require special consideration from treating surgeons. There are currently only nine reported cases of spinal surgery in HCS patients. Herein, we describe the cases of two patients with HCS requiring surgery for progressive spinal deformity. Six months following surgery, both patients reported excellent outcomes with significant improvement in symptoms.

## Introduction

Hajdu-Cheney syndrome (HCS) is a rare disease that was initially described in 1948 by Nicholas Hajdu and Ralphe Kauntze; their patient was a 37-year-old accountant who later died from severe neurological complications [1]. At that time, it was designated a “cranio-skeletal dysplasia.” In 1965, the syndrome was further characterized by William Cheney when he reported the first familial case of a mother with four children who presented with acro-osteolysis [2]. Fewer than 100 total cases have been reported, with minimal descriptions of the spinal findings associated with the disease and implications for treatment.

HCS is most strongly associated with gain-of-function mutations in a transmembrane protein that aids in determining cellular fate and functionality, NOTCH2 [3]. The osteopenia observed in mutant mice with NOTCH2 gain-of-function has been shown to be caused by an increased osteoclast number and induction of receptor activator of nuclear factor kappa-B ligand (RANK-L) production from osteoblasts, though the exact mechanism of underlying bone loss in humans is uncertain [3,4]. HCS can stem from sporadic mutations or maybe familial with autosomal dominant inheritance [4].

Characteristic findings include acro-osteolysis of the distal phalanges (84% of cases) and severe osteoporosis (60% of cases), which is a key finding affecting a wide variety of skeletal groups [5]. While acro-osteolysis is the characteristic finding, severe osteoporosis and fractures may occur in any location in the body and thus patients are predisposed to increased morbidity and mortality. Skeletal malformations may include Wormian bones, open cranial sutures, basilar invagination, premature loss of teeth, midfacial flattening, and short stature. Non-skeletal abnormalities include urinary anomalies, hearing loss, and various cardiac abnormalities. Syringomyelia and upper airway obstruction are also potentially associated conditions that have rarely been described [1,6].

Due to severely osteoporotic bone, spinal deformity progression can be especially pronounced in patients with HCS. This leads to significant morbidity resulting from local cervicomedullary and spinal cord compression. It is uncertain whether operative deformity correction and stabilization provide lasting beneficial outcomes in these patients given the expected skeletal progression.

Below we describe two patients with HCS who presented with signs and symptoms resulting from progressive spinal deformity, with a focus on their clinical course, operative management, and early outcomes. Given the rarity of the condition, we hope this guide helps decision-making for those involved in the care of patients with HCS.

## Case presentation

Case 1

**Figure 1:**
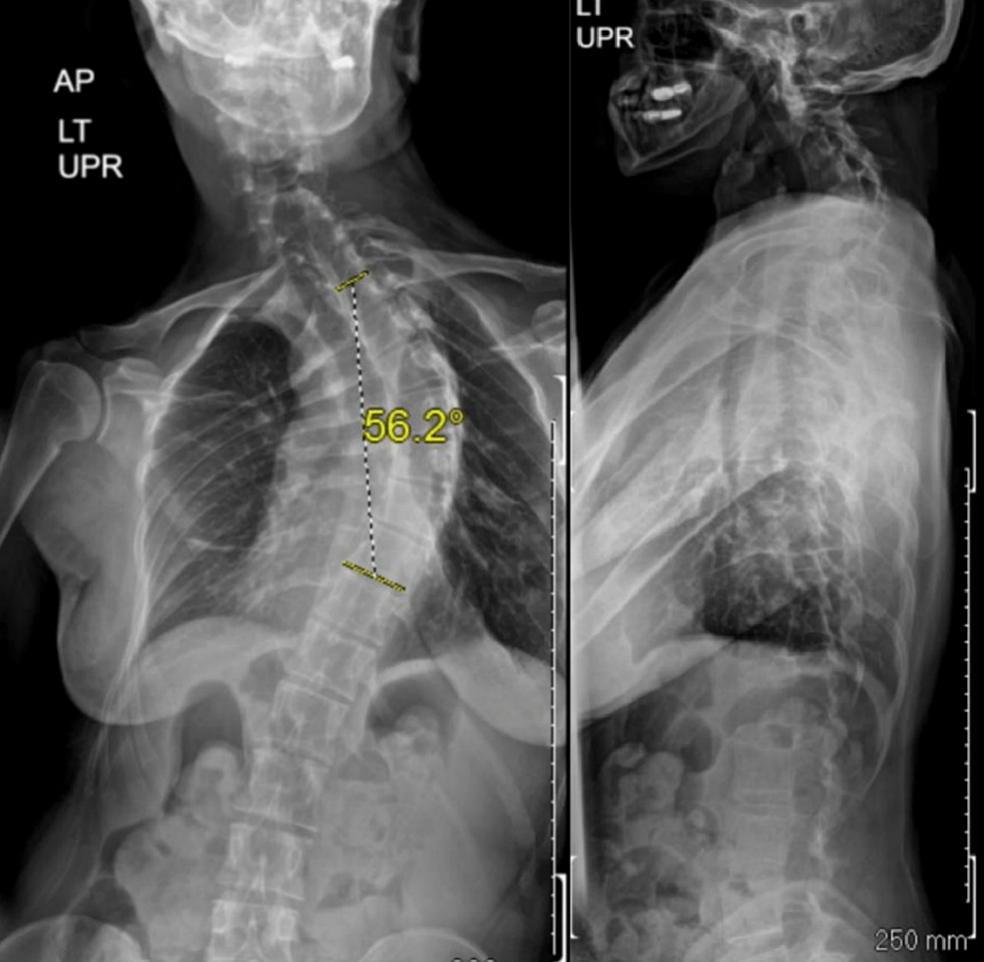
Anterior-posterior and lateral scoliosis plain films demonstrating severe thoracic scoliosis of 56 degrees with thoracic hypokyphosis as well as a compensatory cervical kyphosis.

A 38-year-old woman with HCS presented with severe cervical and upper thoracic back pain that had significantly worsened over the past six months. Over the past several years, she had noted a progressive cervicothoracic deformity. She previously tried physical therapy and other conservative measures with no significant improvement. Her neurological exam was normal, with normal motor or sensory function and no findings of myelopathy; her assessment was otherwise notable for decreased cervical range of motion, significant coronal imbalance, and cervicothoracic scoliosis with associated swan neck deformity and thoracic lordosis. Imaging confirmed these findings (Figure 1), with her thoracic scoliosis measuring 56 degrees and CT findings concerning significant osteoporosis. Flexion-extension plain films demonstrated a fixed deformity. A review of prior imaging revealed continued progression of her deformity over the past several years.

Given these findings, a staged correction was recommended. The first stage of surgery included posterior facetectomies at C4/5, C5/6, and C6/7 with subsequent anterior C5 and C6 corpectomies and C4 to C7 anterior fusion with expandable cage placement and plating. After anterior deformity correction, posterior instrumentation was completed from C4 to C7, intentionally placing her in greater coronal malalignment to allow for global correction with the second stage of surgery. The second stage of surgery was posterior C4-T11 deformity correction and fusion. Bilateral facetectomies were performed from T5 to T8.

**Figure 2:**
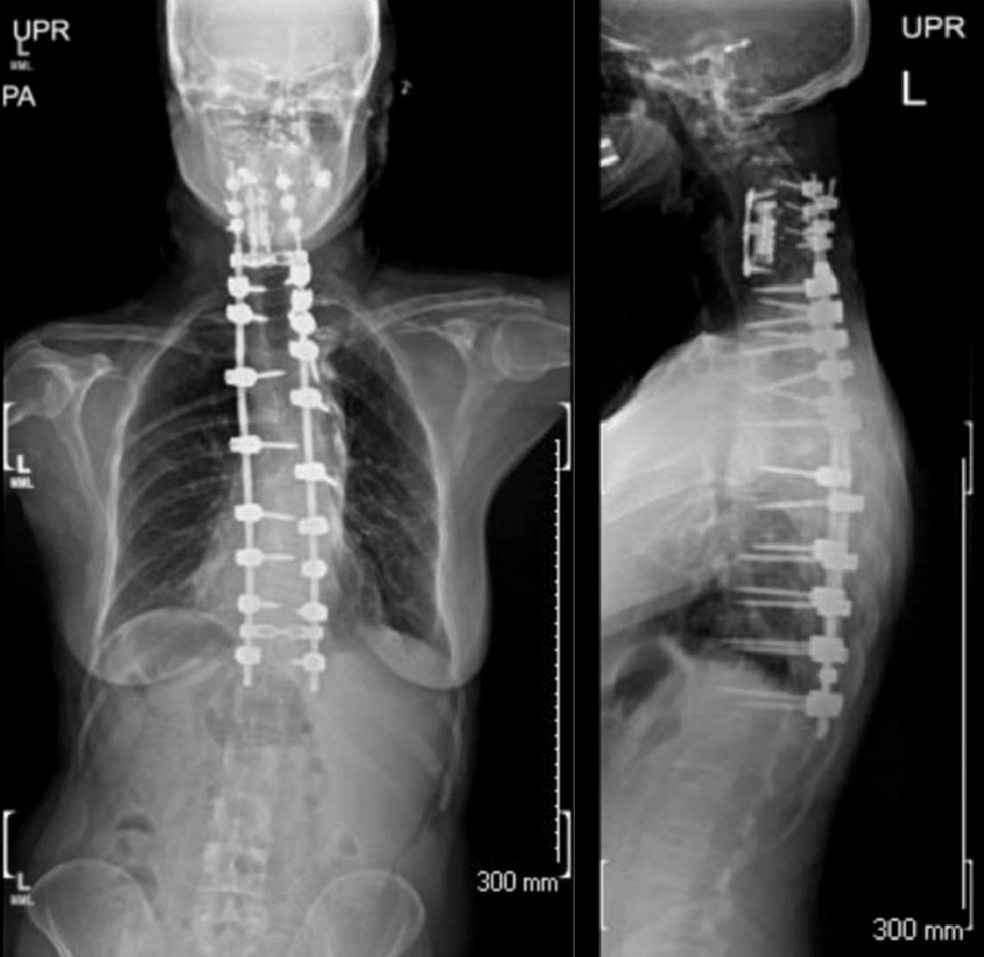
Anterior-posterior and lateral scoliosis plain films demonstrating postoperative changes following anterior C4 to C7 corpectomy and fusion followed by posterior C4 to T11 posterolateral fusion.

Her hospital course was uneventful and she was discharged home on postoperative day six. One month after surgery, she was very happy with her progress; she denied any significant pain and felt that her function was returning appropriately. Plain films showed excellent spinal alignment with no evidence of complications (Figure 2). At six months, she continued to improve with CT imaging demonstrating excellent bony fusion and no fractures. She reported minimal lumbar paraspinal muscle tenderness but was otherwise asymptomatic, with complete resolution of her presenting concerns. Her neurological exam remained normal throughout her perioperative course and follow-up.

Case 2

**Figure 3:**
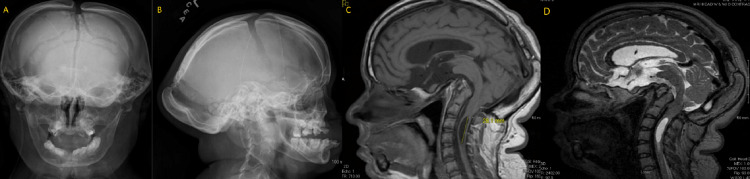
Anterior-posterior and lateral cranial plain films demonstrating significant hyperostosis of the occipital bone as well as nonunion of the cranial sutures (A and B). MRI showing severe basilar invagination with the presence of a cervical syrinx measuring 2.8 cm in length (C and D).

 A 25-year-old woman with HCS and shunted obstructive hydrocephalus presented with headaches, stridulous breathing, and suboccipital neck pain persisting for several years. She also endorsed decreasing balance, unsteadiness, facial numbness/tinging, blurry vision, and subjective hearing loss. She had previously had numerous shunt revisions for the attempted treatment of her symptoms, without improvement. Her physical exam demonstrated a limited cervical range of motion and signs of myelopathy with left-sided hyperreflexia, abnormal Hoffman testing, and clonus. Imaging revealed progressive basilar invagination and associated brainstem compression. An upper cervical syrinx was also present (Figure 3), and this had similarly shown significant progression over several years. Other imaging findings included stigmata associated with HCS, including unfused cranial sutures and changes in her bony facial structures.

Due to the significant nature of her craniocervical instability as well as the progressive worsening of her upper cervical syringomyelia, operative management was recommended. Craniocervical fusion, spanning the cranial vault sutures to C4, was planned. Given dramatic basilar invagination with complex skull base angles relative to her naso-oropharynx, the potential for anterior odontoidectomy was thoroughly discussed, and she elected to defer this with plan for further consideration if the posterior fusion failed to improve her symptoms. Prior to this surgery, an intracranial pressure monitor confirmed normal shunt functioning and lack of correlation between her symptoms and intracranial pressures.

Intraoperatively, the patient was positioned prone; fluoroscopy and neuromonitoring potentials were used to optimize cranial distraction and alignment. A curvilinear sagittal incision, respecting her prior incisions and current shunt position, was connected to a posterior cervical incision to expose from the bilateral frontal bones to C4. Rigid cervical instrumentation and an occipital plate were placed in a standard fashion. Hinged rods from the occipital plate were extended superiorly and contoured to the calvarium with slight reduction of the parietal bones. The rods were secured to the cranial vault using multiple wires via burr holes and cranial mesh. Arthrodesis and fusion of the bilateral frontal bones, parietal bones, occiput, C1, C2, C3, and C4 was completed. Custom craniocervical bracing was continued for six months after surgery.

**Figure 4:**
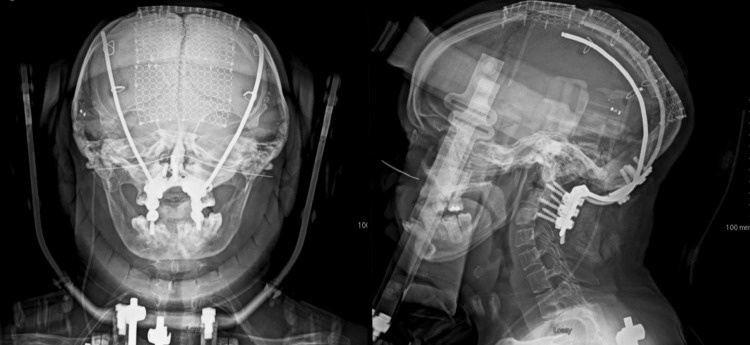
Postoperative AP and lateral plain films demonstrating cranial cervical fusion extending from the bilateral frontal bones to C4. AP, anteroposterior.

Her hospital course was uneventful and she was discharged on postoperative day six. One month after surgery, the patient reported substantial global improvement. The patient felt her balance was improving, her headaches had resolved, and her breathing was much less stridulous. Baseline x-rays were obtained at this visit, which showed good correction of her clival canal angle and appropriate instrumentation. At six months, the patient continued to do well with ongoing complete resolution of her headaches and neck pain. She reported no new neurological symptoms and was very happy with her progress. Her myelopathy had improved. X-rays at this visit demonstrated no signs of complications (Figure 4), and she was successfully transitioned out of her craniocervical orthosis.

## Discussion

Given the low incidence of Hajdu-Cheney syndrome, literature regarding medical and surgical management is limited. There are no high level-of-evidence studies regarding the management of the skeletal manifestations of HCS. Several case reports detail bisphosphonate therapy alone and bisphosphonate therapy with teriparatide in patients with HCS, but the evidence is unclear if these treatments are beneficial and there is no evidence regarding their role in preventing or delaying spinal deformity [7,8].

Patients with HCS have unique surgical considerations. Due to early-onset malalignment and atypical loading of skeletal structures, anatomy may be distorted. For example, a prominent occiput is characteristically present in HCS and is easily visualized in our case. These details require additional considerations and may complicate instrumentation, surgical approach, and provide clues to the underlying pathophysiology. The findings may be explained by combined manifestations of severe osteoporosis and Wolff’s law, which states bony structures remodel in accordance to mechanical loading, and areas of increased compression and tension can show increased density. In addition to Wolff’s law, given the unclear pathophysiology of HCS, it is possible that hyperostosis in these areas of stress may be secondary to the underlying pathophysiology.

In addition to skeletal abnormalities, ligamentous laxity and failure of cranial suture closure in patients with HCS pose a particular challenge for craniocervical surgery. Suture diastasis has been previously reported by Woon and Mardjetko and in their paper labeled “extreme proximal junctional kyphosis” [9]. In patients with significantly patent cranial sutures, a surgical fusion of the sutures should be considered given the potential for devastating neurological outcomes from this complication. While an uncommon extension of the craniocervical fusion to the anterior calvarial vault provides a biomechanical advantage to mitigate the risk of extreme proximal junctional kyphosis.

Perhaps the largest operative concern in patients with HCS, however, is their limited bone quality. For this reason, additional points of fixation, cortical purchase of instrumentation, and locking plates for anterior constructs are encouraged. It is important to note that vertebral anatomy in HCS patients may be suboptimal for safe pedicle screw placement, requiring an extension of the construct to achieve adequate fixation. Bone quality should be optimized with vitamin D and calcium supplementation when appropriate. Multidisciplinary perioperative care with teams specialized in skeletal dysplasia evaluation and management is particularly helpful.

## Conclusions

Hajdu-Cheney syndrome is a rare metabolic bone disorder that results in severe osteoporosis and skeletal abnormalities. Subsequently, patients may have significant morbidity stemming from spinal manifestations. Even slight spinal abnormalities may progress rapidly leading to significant structural deformity. Poor bone quality, anomalous anatomy, and the increased potential for subluxation due to ligamentous laxity are some of the potential factors that must be considered during surgical planning. By adding these cases to the literature, we hope to bring awareness to this condition as well as provide our learned experience to better prepare future practitioners who may encounter this rare condition.
